# A multimodal approach to dementia prevention: A report from the Cambridge Institute of Public Health

**DOI:** 10.1016/j.trci.2015.08.003

**Published:** 2015-10-01

**Authors:** Olawale Olanrewaju, Linda Clare, Linda Barnes, Carol Brayne

**Affiliations:** a Department of Public Health and Primary Care, Cambridge Institute of Public Health, Cambridge, UK; b REACH: The Centre for Research in Aging and Cognitive Health, Department of Psychology, College of Life and Environmental Sciences, University of Exeter University (Professor Linda Clare)

**Keywords:** Aging, Cognitive decline, Dementia, Preventive health, Non-pharmacological intervention, Expert review

## Abstract

**Introduction:**

Globally, dementia is the most frequent form of degenerative condition in the older adult population and poses a major health burden with high socioeconomic costs. So far, attempts to find pharmacologic interventions that can change the onset or progression of dementia have been largely unsuccessful, prompting a shift to focus on interventions aimed at modifying risk factors that occur throughout the life course.

**Methods:**

The Cognitive Function and Ageing Studies, funded by the Medical Research Council, UK, convened three multidisciplinary groups of experts, expert witnesses, and advocates to discuss the state of evidence on primary, secondary, and tertiary dementia prevention and recommend future direction for intervention studies.

**Results:**

Using the United Kingdom Parliamentary Select Committees' approach to gathering evidence, the primary prevention working group focused their deliberation on risk factors strongly associated with dementia. The group highlighted the need for high-quality studies to assess the effects of behavioral intervention on the delay of cognitive decline and dementia onset.

**Discussion:**

The working group recommended that the development of a future dementia prevention trial should use a multimodal, multifactor, multilevel, community and individually tailored approach.

## 1 Background

Globally, dementia is the most frequent form of degenerative condition in the older adult population and poses a major health burden with high socioeconomic costs [1]. In 2010, 35 million people were estimated to be living with dementia worldwide at a cost of over 600 billion USD [1]. Attempts to find a cure have been largely unsuccessful, prompting a shift to focus on interventions aimed at modifying risk factors that occur throughout the life course [2], [3], [4], [5]. The Cambridge Institute of Public Health is one member of ongoing international collaborations committed to repurposing existing population cohort studies of late middle age to older age groups to investigate dementia prevention. Such international collaborative projects to date include EURODEM, 21st Century EURODEM, EU–HATICE (Healthy Ageing Through Internet Counselling in the Elderly, www.hatice.eu), The European Dementia Prevention Initiative, and European Prevention of Alzheimer's disease. In this article, we present findings and recommendations from the dementia primary prevention working group based at the Institute and conclude with a consideration of challenges facing the design of prevention trial design.

## 2 Methods

Between December 2013 and May 2014, the Cognitive Function and Ageing Studies (CFAS), funded by the Medical Research Council, UK (MRC), convened three multidisciplinary groups of experts, expert witnesses, and advocates to discuss the state of evidence on primary, secondary, and tertiary prevention of dementia and recommend future directions for intervention studies [6]. These workshops were conceived from a series of deliberations by a national collaboration of UK-based institutions to evaluate the current state of knowledge on a dementia prevention and intervention strategy. The CFAS are large UK-based longitudinal multicentre studies looking at health and cognitive decline in those who are aged >65 years [6]. The MRC-CFAS Prevention Working Groups (CFAS-PWG) are multidisciplinary and composed of experts and advocates in the dementia research field, including representation from disciplines such as epidemiology, biostatistics, psychiatry, neuropathology, psychology, social sciences, and translational medicine (Supplementary Data). The primary prevention working group adopted the method of the Select Committees of the Houses of Parliament in the UK. This included a witness approach with requests for written and verbal evidence, Skype, or in-person interview where possible on a particular topic. Information gathered from witnesses was transcribed.

## 3 Summary of evidence

Epidemiological studies have reported consistent findings on modifiable risk and protective factors associated with the development of dementia [7], [8], [9], [10], [11]. Many of these risk factors are shared with other noncommunicable conditions (cardiovascular diseases, cancer, and diabetes), for which cost-effective preventive interventions are currently sought [12]. Therefore, the CFAS-PWG focused their deliberation on risk factors most strongly associated with dementia, namely cognitive activity, physical activity, social engagement, diet and nutrition, medication optimization, vascular risk reduction, mental health, and stress. Most evidence for associations comes from observational studies, with a small number of randomized controlled trials presenting evaluations of intervention approaches, often with significant quality limitations [13].

### 3.1 Single-domain intervention

The evaluation of single-component interventions in the prevention of cognitive decline and dementia presents a mixed picture, with a few positive results and many studies proving ineffective [14], [15], [16], [17]. The benefits of physical activity for noncognitive aspects of health and quality of life in the older population are well documented, but there is limited evidence on the effect of physical activity in delaying the onset of cognitive decline or dementia onset [16], [18]. Systematic reviews of intervention studies suggest that strengthening exercises, aerobic exercises, or a combination of these show promising preventative effects on cognitive decline in older people [19], [20]. However, many of the primary studies reviewed lack statistical power and their results are inconclusive [20], [21]. The effect of cognitive training on the trajectory of cognitive decline and dementia is also unclear [17], [18]. Although some studies show promising results, more rigorous trials are still needed [22], [23].

Cardiovascular risk factors including hypertension and hypercholesterolemia, together with diabetes mellitus and obesity, are associated with increased risk of dementia, whereas treatment of hypertension and hypercholesterolemia is associated with a decrease in incident dementia [24]. Although there is some evidence that controlling hypertension and systolic blood pressure could be associated with preventing cognitive decline in midlife, there is limited evidence on the effect of treating vascular risk factors to prevent cognitive impairment or dementia in late-life [25], [26], [27]. There is a dearth of quality studies on the effects of interventions addressing medication management, social engagement, and lifestyle factors such as smoking cessation and altered alcohol intake on the risk of developing dementia, although observational studies have demonstrated that these factors are associated with dementia risk [4], [10], [11], [28], [29].

A systematic review of intervention studies evaluating whether diet or nutritional factors can help to prevent cognitive decline found that omega-3 (n-3) polyunsaturated fatty acids supplementation might be effective in the prevention of dementia [30]. The evidence on the effect of antioxidants and B vitamin supplements is less promising, while the effects of healthy diets such as the Mediterranean diet need to be confirmed in intervention studies [30], [31], [32], [33]. There is good evidence to show that behavioral interventions addressing potentially modifiable lifestyle risk factors offer the possibility of reducing risk of cognitive decline, but CFAS-PWG acknowledges that there is as yet insufficient evidence from high-quality trials to permit a firm conclusion on their effects.

### 3.2 Multimodal intervention

Dementia is a complex syndrome that affects a heterogeneous population and carries various risk factors. Therefore, trials which target multiple domains in combination are likely to be more effective than those that use single-component interventions [3]. To date, only a few multidomain or multi-intervention trials have evaluated the effects of behavioral interventions on dementia prevention. There are three ongoing large dementia prevention trials in Europe. The Prevention of Dementia by Intensive Vascular Care study is a cluster randomized controlled trial (RCT) evaluating the effect of multicomponent nurse-led vascular care versus usual care in the nondemented middle-old [34]. The primary outcomes are incident dementia and disability. The Multidomain Alzheimer Preventive Trial is a RCT aimed at evaluating the effects of multicomponent intervention on change in cognitive function in the middle- to old-old with subjective memory complaints [35]. The Finnish Geriatric Intervention Study to Prevent Cognitive Impairment and Disability (FINGER) evaluates the effect of interventions delivered using a combination of individual and group sessions on cognition in at-risk young- to middle-old [36].

The FINGER 2-year results found a positive effect of the multicomponent intervention on change in cognitive function. Estimated mean change in neuropsychological test battery (NTB) total z-score at 2 years was 0.2 (standard error [SE], 0.01; standard deviation [SD], 0.51) in the intervention group and 0.16 (SE, 0.01; SD, 0.51) in the control group [37]. FINGER also found that after 2 years, participants in the control group increased their risk of cognitive decline compared with those in the intervention group (NTB, odds ratio, 1.31; 95% confidence interval, 1.01–1.71). However, the overall effects of multimodal interventions need to be disentangled to understand the contribution of individual components. Given the available evidence on multiple treatment interactions and effects, the CFAS-PWG experts recommended that a multimodal intervention approach addressing the aforementioned domains is likely to be more effective compared with single-domain treatment.

## 4 Discussion

### 4.1 Multilevel, multicomponent, and individually tailored

Due to the complexity of dementia prevention, the CFAS-PWG recommend that dementia trial designs should be tailored to the needs of individuals and communities and the relevant risk factors, and both initiated and evaluated at individual, household, and community levels. The heterogeneity of the older population based on risk factors and age and possibly biomarkers should be considered explicitly, with the goal of finding the best intervention for individuals defined by these characteristics [2]. The target participant groups for preventive efforts would range from the healthy and optimally functioning to those with multiple health problems and those with cognitive impairment and dementia.

Individual-level interventions should focus on ways to optimize and maintain healthy functioning. A modular approach may be taken to address different risk and protective factors, acknowledging that participants' needs will differ. For example, some people might benefit from simple provision of information, whereas others might need a more intensive approach. Having people actively identify what they need to or are willing to do is also important. For example, a community facilitator could signpost people to particular resources or activities. Here again, the comparison might be between an active goal-setting approach and simple provision of information. Furthermore, individual-level interventions for the high-risk groups could include a HATICE-style intervention combined with medication management.

A household-level intervention would ideally involve working with everyone in the household to identify environmental modifications and other changes that could be made. Practical examples include taking televisions out of the bedrooms, removing remote controls to promote movement, or introducing smaller plates to reduce portion size. The CFAS-PWG group recognizes that it is important not to be seen as imposing changes, but rather to help people to internalize or “own” the changes they wish to make. Finally, community-level interventions would involve working with local authorities to identify localities (streets, neighborhoods, or deprivation areas) where initiatives can be developed to encourage more physical and cognitive activity and social engagement. The CFAS-PWG group recommends that a cluster RCT would be most appropriate form of evaluation for these interventions.

### 4.2 Other challenges

The CFAS-PWG highlighted the need for quality studies to assess the effects of behavioral intervention on the delay of cognitive decline and dementia onset. To achieve this, study designs need to be RCTs or adaptive trials, have larger samples, cover a sufficiently long follow-up period, and identify the most appropriate outcome measures [13]. As prevention of dementia would be a distal outcome, it is important to focus on a range of behavioral outcomes demonstrating changes that may be associated with reduced risk. For example, the FINGER study has shown that change in cognitive function is a sensitive enough measure for use as a proximal and a distal outcome in future trials [37]. It is also important to consider what factors will motivate participants to engage in interventions. Offering the opportunity to improve health and well-being in the short to medium term may be more engaging and motivating than offering interventions to reduce the risk of developing dementia in the future. In addition to agreeing on standardized outcomes for dementia prevention, there is a further need to form a consensus on the optimal “dose” or intensity of interventions required to detect significant effects [18]. Failing this, research in this area could be open to multiple interpretations with the risk of failing to detect positive effects of potentially worthwhile approaches [18]. Other practical challenges cited by the CFAS-PWG included securing funding, designing affordable and translatable interventions, and how to best collaborate across countries and studies.

## 5 Conclusion

The limited evidence available about reducing the risk of developing dementia, coupled with the strong and growing interest in this topic, presents an important opportunity. The CFAS Dementia Primary Prevention Working Group looked at evidence available at the time of meeting (2014) and proposed a holistic approach to designing a dementia prevention trial. A summary of the group's evidence review is presented using an evidence synthesis matrix (Table 1). The work embodied several key principles including sustainability, feasibility, potential for wider implementation at community and household levels, and an individual perspective. The core recommendation is that the development of future dementia prevention trials should use a multimodal, multifactor, multilevel, as well as individually tailored approaches.

**Table 1:** Primary prevention working group evidence synthesis matrix

Modality	Cognitive activity—thinking	Physical activity—moving	Social engagement—mixing	Diet and nutrition—eating and drinking (including other lifestyle factors)	Medication optimisation	Physical health (vascular risk reduction)	Mental health—mood	Well-being—stress reduction
Individual in-person	Evidence from RCTs of specific cognitive training or cognitive activity [17]. Some studies provide evidence of benefits to cognition. Advanced Cognitive Training for Independent and Vital Elderly Study [23] provides evidence of generalisation to everyday functioning. Quality of evidence generally low.	Evidence from RCTs of physical exercise interventions. Some studies find benefits to cognitive function [19], [38]. Quality of evidence generally low.	No evidence from intervention trials.	Evidence from RCTs suggests that adherence to Mediterranean diet may reduce risk but dietary supplementation is not effective [30]. Quality of evidence generally poor. No direct evidence that smoking cessation delays or prevents dementia. No evidence from intervention trials regarding alcohol intake. MAPT to report soon [35].	No evidence that particular medications protect against or prevent dementia. Evidence for harmful effects of polypharmacy from observational studies.	Limited evidence to date but PreDIVA trial will report in 2015 [34]	One study suggests that long-term treatment with tricyclics or monoamine oxidase inhibitors (but not selective serotonin reuptake inhibitors) may reduce incidence of dementia [39].	No evidence from intervention trials.
Internet based	Internet based or computerized interventions are regarded as offering promising effects.	—	—	—	—	—	—	—
Immediate network/community	Some cognitive activity interventions at this level report benefits to cognition [40].	Environmental modification approaches suggested—no research evidence. Some activity-based interventions operate at this level.	Some activity-based interventions at this level include social support/social networks.	Environmental modification approaches suggested – no research evidence.	—	—	—	—
Wider community	—	—	—	Policy interventions suggested—no research evidence	—	—	—	—

Abbreviations: RCT, randomized controlled trial; MAPT, MultiDomain Alzheimer Preventive Trial; PreDIVA, Prevention of dementia by intensive vascular care.

NOTE. The matrix table is populated with a brief summary of evidence considered by the Primary Prevention Working group. The rows represent potential areas of delivery for interventions, whereas the entries in the columns give a summary of evidence for each mode of intervention.

**Research in context**

1. Systematic Review: This was an expert review by the MRC-CFAS Prevention Working Groups (CFAS-PWG), which gathered evidence using the method of the Select Committees of the Houses of Parliament in the United Kingdom. This included a witness approach with requests for written and verbal evidence, or Skype or in person interview where possible on a particular topic. Much of the evidence used during deliberation was compiled using available systematic reviews, supplemented where appropriate by opinion from narrative reviews and by details of individual studies. Individual studies were either already known or were identified by expert witnesses.
2. Interpretation: This review highlighted the need for high quality studies to assess the effects of behavioural intervention on the delay of cognitive decline and dementia onset.
3. Future Directions: The working group recommended that the development of future dementia prevention trial should use a multi-modal, multi-factor, multi-level, and individually tailored approach.
