# Supplementary material for: A multimodal approach to dementia prevention: A report from the Cambridge Institute of Public Health
Source: Alzheimers Dement (N Y). 2015 Oct 1;1(3):151–6. doi: 10.1016/j.trci.2015.08.003 (PMC4750875; doi:10.1016/j.trci.2015.08.003)
Supplement: Supplementary Data [file mmc1.docx]

## Appendix 1: Members of the Dementia Prevention Working Groups, Expert Witnesses and Advocates

**Primary Prevention group Secondary Prevention group**

Professor Carol Brayne Professor. Craig Ritchie

Professor Linda Clare Dr. Blossom Stephan

Cherie McCracken – now retired. Dr. George Savva

David Melzer Dr. Chris Fox

Dr. Alesandro Ble Professor Simon Lovestone

Dr. Martin Knapp Professor Tony Arthur

Dr. Louise Lafortune

**Tertiary Prevention group Design/Methodology group**

Professor John O’Brien Dr. Fiona Matthews

Professor Tom Dening Dr. Adrian Mander

Professor Rob Howard

Professor Alan Thomas **ELSI group**

Dr. Richard Perry Professor Bronwyn Parry

Professor Rowan Harwood Dr. Shirlene Badger

Dr. Liz Sampson Denise Wilson

Professor Ian McKeith Richard Milne

Professor Bob Woods Cathy Baldwin

Professor Louise Robinson Simon Harrison

Professor Gill Manthorpe Linda Barnes

**International Panel**

Dr. Edo Richard - HATICE

**Expert witnesses**

Kaarin Anstey – **Risk reduction through modular internet-based lifestyle interventions**

Simon Griffin **– Physical activity**

Sarah Hilmer – **Modification of medication**

Ian Rees Jones – **Health implications of social inequality and diversity**

Nicola Lautenschlager – **Physical activity interventions**

Theresa Marteau – **Behaviour change**

Susan Michie – **Behaviour change**

David Ogilvie – **Physical activity and built environment**

George Rebok – **ACTIVE and Experience Corps**

Ian Robertson – **Cognition-focused intervention**

Michael Valenzuela – **Cognitive reserve and cognition-focused intervention**

Christina Victor – **Social engagement and well-being**

Advocates:

Age UK – **Susan Davidson**

**Alzheimer’s Society representative**

Blue Zones – **Dan Buettner**

**Campaign to End Loneliness representative**
